# Prognostic significance of NFIA and NFIB in esophageal squamous carcinoma and esophagogastric junction adenocarcinoma

**DOI:** 10.1002/cam4.1434

**Published:** 2018-03-25

**Authors:** Bo Yang, Zhi‐hang Zhou, Li Chen, Xiang Cui, Jun‐yan Hou, Kai‐jie Fan, Si‐hao Han, Peng Li, Shao‐qiong Yi, Yang Liu

**Affiliations:** ^1^ Department of General Thoracic Surgery The General Hospital of PLA Beijing China; ^2^ Department of Digestive Disease the Second affiliated hospital of Chongqing Medical University Chongqing China; ^3^ Department of Emergency The General Hospital of PLA Beijing China; ^4^ Department of Orthopedics The General Hospital of PLA Beijing China; ^5^ The Medico‐technical Division The General Hospital of PLA Beijing China; ^6^ Harvard T.H.Chan School of Public Health Boston Massachusetts; ^7^ Department of General Surgery The General Hospital of PLA Beijing China

**Keywords:** Esophageal squamous cell carcinoma, esophagogastric junction adenocarcinoma, NFIA, NFIB, prognosis

## Abstract

The nuclear factor I (NFI) family members, especially NFIA and NFIB, play essential roles in cancers. The roles of NFIA and NFIB in esophageal squamous cell carcinoma (ESCC) and esophagogastric junction adenocarcinoma (EJA) remain poorly known. This study aimed to determine the expression of NFIA and NFIB in ESCC and EJA and elucidate their prognostic significance. The expression of NFIA and NFIB was examined in 163 ESCC samples and 26 EJA samples by immunohistochemistry. The results showed that high NFIA expression correlated significantly with poor differentiation, lymph node metastasis, and advanced TNM stage in patients with ESCC. High NFIB expression only correlated with poor differentiation in patients with ESCC. Survival analysis showed that NFIA but not NFIB associated with short overall survival (OS) and disease‐free survival (DFS) of patients with ESCC. On the other hand, high NFIB expression correlated with lymph node metastasis, advanced TNM stage, and short OS and DFS in patients with EJA. Finally, multivariate analysis demonstrated that high NFIA expression was an independent prognostic factor for ESCC. Taken together, these results demonstrated that NFIA and NFIB could serve as prognostic indicators for ESCC and EJA, respectively.

## Introduction

Esophageal squamous carcinoma (ESCC) accounts for more than 90% of esophageal cancer (EC), which is one of the most prevalent cancers in Africa and Asia 1. Generally, ESCC is diagnosed at late stages and the prognosis is poor despite the application of multidisciplinary therapy. Most patients die within 1 year after diagnosis, and the five‐year survival rate is only 8% to 20% 2. Esophagogastric junction adenocarcinoma (EJA) is defined as the carcinoma that across the esophagogastric junction line, including both distal esophageal adenocarcinoma and proximal gastric cancer 3. Accumulating studies reveal that EJA is different from gastric and esophageal adenocarcinoma in molecular features, pathological evolution, and clinical behavior 4. The incidence of EJA has risen fast in North America, Europe, and East Asia over the last two decades 5. The five‐year survival rate of EJA is as low as 10–15% 6. Revealing novel molecular markers is urgently needed to improve the prognosis of patients with ESCC and EJA.

The nuclear factor I (NFI) family, initially found to function in adenoviral DNA replication, consists of four genes (*NFIA*,* NFIB*,* NFIC*, and *NFIX*). These genes encode nuclear factors that bind to TTGGC(N5)GCCAA sequence as homo‐ or heterodimers to activate or suppress gene transcription depending on the cellular context and regulatory region 7. The NFIs were then demonstrated to play crucial roles in the development of many organ systems such as the central nervous system and lung 8. Recent studies revealed that NFIs, especially NFIA and NFIB, also function in the development or progression of cancers 7. In contrast to the clear role of NFIA as a tumor‐promoting gene in glioma 9 and esophageal carcinoma 10, the role of NFIB in carcinogenesis or progression is context‐dependent 11. On the one hand, NFIB acts as a tumor‐promoting gene in small‐cell lung cancer (SCLC) 12, 13, 14, 15, melanoma 16, breast cancer 17, 18, and colon cancer 19. On the other hand, NFIB acts as a tumor suppressor in other cancers including osteosarcoma 20, cutaneous squamous cell carcinoma 21, and non–small‐cell lung cancer (NSCLC) 22. However, the expression and clinicopathological value of NFIA and NFIB in ESCC and EJA are yet to be explored.

This study aimed to elucidate the prognostic value of NFIA and NFIB in 163 patients with ESCC and 26 patients with EJA using immunohistochemistry. The results showed that high NFIA expression correlated with poor differentiation, lymph node metastasis, and short overall survival (OS) and disease‐free survival (DFS) in patients with ESCC. High NFIB but not NFIA expression correlated with poor differentiation, lymph node metastasis, and short OS and DFS time in patients with EJA. These results demonstrated the distinct roles of NFIA and NFIB in esophageal cancer.

## Materials and Methods

### Patients and primary tissue samples

Esophageal squamous cell carcinoma (*n* = 163) and esophagogastric junction adenocarcinoma (*n* = 26) tissues were obtained from 189 patients who underwent esophagectomy resection with lymph node dissection during the period from 2012 to 2015 at the PLA General Hospital and the 309th Hospital of PLA. The cancer tissues and corresponding paracancerous tissues were applied to produce human tissue microarray (3 cores/tissue). The criteria for selecting patients were as follows: (1) did not have synchronous tumors or multiple metachronous tumors; and (2) did not receive preoperative chemotherapy or radiation therapy. The samples were embedded in paraffin after 24 h of formalin fixation. The diagnoses of esophageal squamous cell carcinoma and esophagogastric junction adenocarcinoma were made independently by at least two pathologists. Staging was principally based on the eighth staging primer of esophagus and esophagogastric junction cancer 23. All the patients gave informed consent (written) before research. This study was carried out in accordance with the principles of the Helsinki Declaration and approved by the Ethical Committee of the PLA General Hospital.

### Immunohistochemistry

Immunohistochemistry was performed as previously described 24. Briefly, after being deparaffinized and rehydrated, the sections were boiled in 10 mmol/L citrate buffer (pH 6.0) for 15 min in a microwave oven. The sections were then incubated with anti‐NFIA (1:100, catalog no. GR195242‐1; Abcam, Cambridge, MA) or NFIB antibodies (1:100, catalog no. GR229339‐13; Abcam) overnight at 4°C. Sections were washed for one hour in TBST and then incubated with a secondary antibody (DAKO, Denmark) at a dilution of 1:100 in TBST. Finally, the sections were visualized using diaminobenzidine solution (DAKO Denmark). Sections without incubation with primary antibody served as negative controls.

### Evaluation of immunostaining results

The intensity of staining (brown color) was semiquantitatively scored as follows: 1, weak; 2, medium; 3, strong; and 4, very strong. The percentage of maximally stained tumor cells in each section was recorded (0, <5%; 1, 5–30%; 2, 30–50%; 3, >50%). The intensity of the staining multiplied by the percentage of positive cells yields the combined score of a sample. High expression of NFIA/NFIB was defined as a combined score for the intensity and area of staining that was larger than 3, which is determined by the X‐tile software (Rimm Lab, Yale University, New Haven, CT). The results were verified by two pathologists independently.

### Statistical analyses

Kolmogorov–Smirnov test was used to estimate the normality of distributions. Statistical significance was analyzed with SPSS 18.0 software (SPSS, Chicago, IL). The correlation between NFIA or NFIB and clinicopathological features was analyzed by chi‐square test. Differences in noncategorical variables between subgroups were tested with the nonparametric Mann–Whitney *U*‐test. The OS and DFS were calculated from the date of surgery to the date of the final follow‐up or event using the Kaplan–Meier method. The survival curve was assessed by the log‐rank test. Univariate Cox analysis was applied to evaluate the association between the clinicopathological parameters, NFIA/NFIB expression, and patients’ survival. Multivariate Cox proportional hazards regression model was further used to investigate the independent prognostic factors. *P* values less than 0.05 were considered statistically significant.

## Results

### High NFIA expression correlated with lymph node metastasis and poor differentiation in ESCC

**Figure 1:**
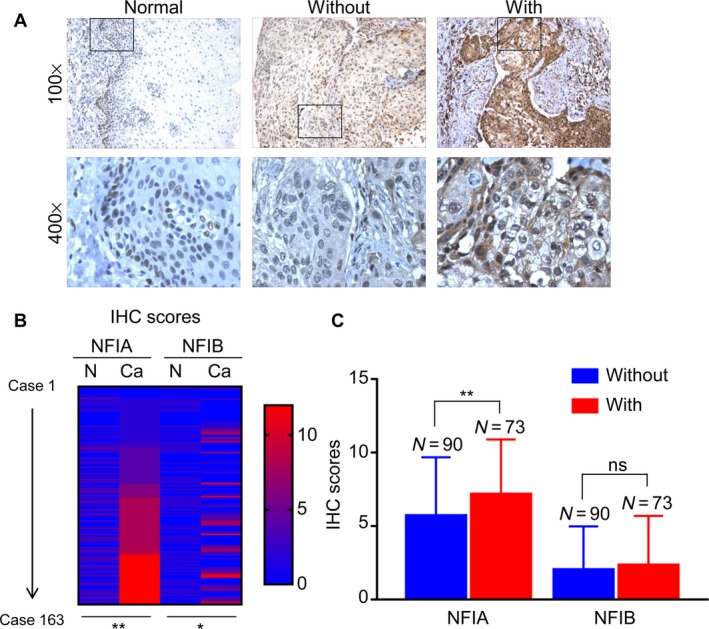
High NFIA expression correlates with lymph node metastasis in esophageal squamous cell carcinoma (ESCC). (A) Representative images showing the expression of NFIA in normal esophageal epithelia and ESCC tissues with or without lymph node metastasis. (B) Heat map showing the IHC scores of NFIA and NFIB in ESCC tissues and corresponding normal esophageal epithelia. (C) IHC scores of NFIA in ESCC tissues with or without lymph node metastasis. *P < 0.05; **P < 0.01.

**Figure 2:**
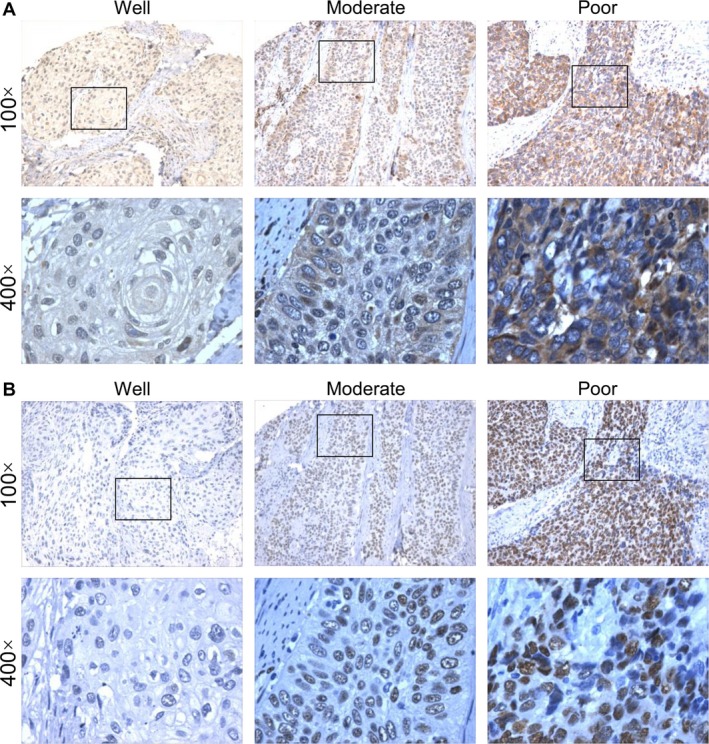
High NFIA expression correlates with poor differentiation in esophageal squamous cell carcinoma (ESCC). (A) Representative images showing the expression of NFIA in ESCC tissues with good, moderate, or poor differentiation. (B) Representative images showing the expression of NFIB in ESCC tissues with good, moderate, or poor differentiation.

The expression of NFIA and NFIB was firstly evaluated in ESCC tissues from 163 patients, including 135 males and 28 females. The average age at diagnosis was 60.9. As shown in Figure 1A, NFIA and NFIB were expressed if any only in basal cells of normal esophageal epithelia, mainly located in the nucleus, while in ESCC tissues, NFIA was highly expressed in cancer cells, located in both the nucleus and cytoplasm. High expression of NFIA was found in cancerous tissues from 104 patients (63.8%). The expression of both NFIA and NFIB was significantly higher than that in normal esophageal epithelia (Fig. 1B). Chi‐square test revealed that high NFIA expression significantly correlated with poor differentiation (*P *=* *0.046), lymph node metastasis (*P *=* *0.021), and advanced TNM stage (*P *=* *0.045) in ESCC, while high NFIB expression only correlated with poor differentiation degree (*P *=* *0.038) (Table 1). NFIA expression was higher in cancer tissues with lymph node metastasis than in those without lymph node metastasis (Fig. 1C). The expression of NFIA and NFIB in cancer tissues with different differentiation degree is shown in Figure 2A and B, respectively. It is obvious that both NFIA and NFIB were highly expressed in poorly differentiated ESCC. Taken together, these results revealed that NFIA was highly expressed in ESCC tissues and high NFIA expression correlated with poor differentiation, lymph node metastasis, and advanced TNM stage in ESCC.

**Table 1:** Correlation between NFIA/NFIB expression and clinicopathological features in cancer tissues from 163 patients with esophageal squamous cell carcinoma

Clinicopathologic features	No. of patients (%)	NFIA expression status		*P* value	NFIB expression status		*P* value
		Low (*n* = 59) No. of patients (%)	High (*n* = 104) No. of patients (%)		Low (*n* = 110) No. of patients (%)	High (*n* = 53) No. of patients (%)	
Gender							
Male	135 (82.8)	49 (36.3)	86 (63.7)	1.000	91 (67.4)	44 (32.6)	1.000
Female	28 (17.2)	10 (35.7)	18 (64.3)		19 (67.9)	9 (32.1)	
Age							
≤60	74 (45.4)	27 (36.5)	47 (63.5)	1.000	49 (66.2)	25 (33.8)	0.867
>60	89 (54.6)	32 (36.0)	57 (64.0)		61 (68.5)	28 (31.5)	
Tumor size (cm)							
≤4.0	99 (60.7)	37 (37.4)	62 (62.6)	0.741	68 (68.7)	31 (31.3)	0.733
>4.0	64 (39.3)	22 (34.4)	42 (65.6)		42 (65.6)	22 (34.4)	
Differentiation degree							
Well	40 (24.5)	21 (52.5)	19 (47.5)	**0.046**	33 (82.5)	7 (17.5)	**0.038**
Moderate	66 (40.5)	21 (31.8)	45 (68.2)		44 (66.7)	22 (33.3)	
Poor	57 (35.0)	17 (29.8)	40 (70.2)		33 (57.9)	24 (42.1)	
T‐stage							
T1+T2	45 (27.6)	17 (37.8)	28 (62.2)	0.856	27 (60.0)	18 (40.0)	0.262
T3+T4	118 (72.4)	42 (35.6)	76 (64.4)		83 (70.3)	35 (29.7)	
Lymph node metastasis							
Negative	90 (55.2)	40 (44.4)	50 (55.6)	**0.021**	63 (70.0)	27 (30.0)	0.503
Positive	73 (44.8)	19 (26.0)	54 (74.0)		47 (64.4)	26 (35.6)	
Distant metastasis							
Negative	163 (100.0)	59 (36.2)	104 (63.8)	na	110 (67.5)	53 (32.5)	na
Positive	0 (0)	0 (0)	0 (0)		0 (0)	0 (0)	
TNM stage							
I	12 (7.4)	2 (16.7)	10 (83.3)	**0.045**	6 (50.0)	6 (50.0)	0.363
II	91 (56.4)	40 (44.0)	51 (56.0)		64 (70.3)	27 (29.7)	
III	60 (36.8)	17 (28.3)	43 (71.7)		40 (66.7)	20 (33.3)	

Chi‐square test was used to evaluate the correlation between NFIA/NFIB expression and clinicopathological features. The bold values indicated that the *P* value was smaller than 0.05.

### High NFIA expression is an independent predictor of poor prognosis in patients with ESCC

**Figure 3:**
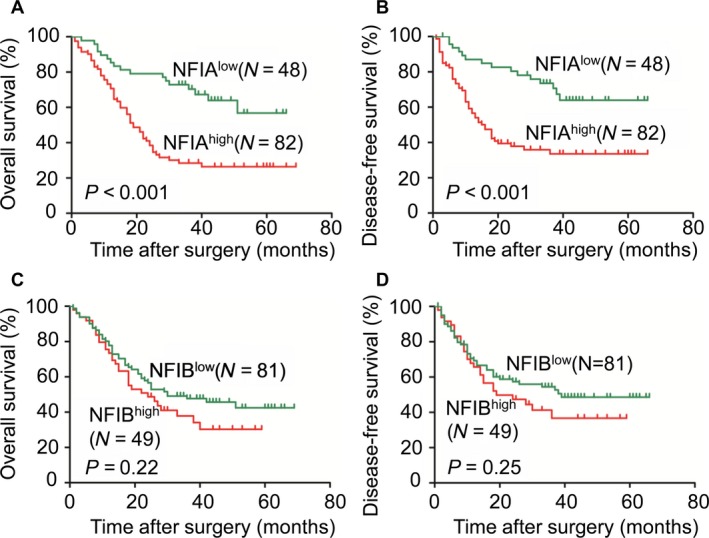
High NFIA expression is a predictor of poor prognosis in patients with esophageal squamous cell carcinoma (ESCC). (A and B) The Kaplan–Meier survival analysis showing that ESCC patients with high NFIA expression tend to have a shorter OS (A) or DFS (B) time. (C and D) The Kaplan–Meier survival analysis showing that NFIB expression was correlated with neither OS (C) nor DFS (D) time.

We obtained follow‐up information from 130 patients with ESCC, including 111 males and 19 females. The mean follow‐up time was 27.9 months. The Kaplan–Meier analysis illustrated that high NFIA expression correlated with short OS (Fig. 3A; *P *<* *0.001) or DFS (Fig. 3B; *P *<* *0.001) time in patients with ESCC, but NFIB did not correlate with OS or DFS time (Fig. 3C and D). Univariate Cox regression analysis showed that high NFIA expression (hazard ratio (HR) = 3.031, 95% confidence interval (CI) = 1.754–5.239, *P *<* *0.001), tumor size (HR = 1.781, 95% CI = 1.131–2.805, *P *=* *0.013), T‐stage (HR = 2.334, 95% CI = 1.304–4.179, *P *=* *0.004), and lymph node metastasis (HR = 3.660, 95% CI = 2.661–5.925, *P *<* *0.001) were prognostic risk factors for OS (Table 2). Besides, high NFIA expression (HR = 3.044, 95% CI = 1.697–5.457, *P *<* *0.001), T‐stage (HR = 2.156, 95% CI = 1.173–3.963, *P *=* *0.013), and lymph node metastasis (HR = 4.116, 95% CI = 2.442–6.936, *P *<* *0.001) were prognostic risk factors for DFS (Table 2). Multivariate Cox proportional hazards regression analysis revealed that high NFIA expression (HR = 3.450, 95% CI = 1.908–6.240, *P *<* *0.001), lymph node metastasis (HR = 2.636, 95% CI = 1.565–4.439, *P *<* *0.001), and T‐stage (HR = 2.272, 95% CI = 1.224–4.217, *P *=* *0.009) were independent risk factors for OS in ESCC (Table 3). High NFIA expression (HR = 3.388, 95% CI = 1.801–6.371, *P *<* *0.001), lymph node metastasis (HR = 3.628, 95% CI = 2.020–6.517, *P *<* *0.001), and T‐stage (HR = 2.228, 95% CI = 1.166–4.256, *P *=* *0.015) were also independent risk factors for DFS in ESCC (Table 3). These results demonstrated that high NFIA expression is an independent prognostic factor in ESCC.

**Table 2:** Univariate Cox regression analysis of the risk factors in esophageal squamous cell carcinoma

Clinicopathologic features	OS			DFS		
	HR	95% CI	*P* value	HR	95% CI	*P* value
Gender (female/male)	0.603	0.290–1.256	0.177	0.697	0.332–1.463	0.340
Age (>62/≤62)	1.177	0.743–1.864	0.489	1.193	0.728–1.955	0.483
Tumor size (cm) (>4.3/≤4.3)	1.781	1.131–2.805	**0.013**	1.435	0.880–2.340	0.148
Differentiation degree (Well/Moderate/Poor)	1.027	0.763–1.384	0.860	0.934	0.678–1.285	0.675
T‐stage (T3 + T4/T1 + T2)	2.334	1.304–4.179	**0.004**	2.156	1.173–3.963	**0.013**
Lymph node metastasis (positive/negative)	3.660	2.261–5.925	**0.000**	4.116	2.442–6.936	**0.000**
NFIA (high/low)	3.031	1.754–5.239	**0.000**	3.044	1.697–5.457	**0.000**
NFIB (high/low)	1.333	0.840–2.114	0.222	1.328	0.809–2.179	0.262

OS, overall survival; DFS, disease‐free survival. The bold values indicated that the *P* value was smaller than 0.05.

**Table 3:** Multivariate Cox regression analysis of the risk factors in esophageal squamous cell carcinoma

Clinicopathologic features	OS			DFS		
	HR	95% CI	*P* value	HR	95% CI	*P* value
Gender (female/male)	0.765	0.356–1.640	0.491	0.877	0.405–1.900	0.740
Age (>62/≤62)	1.331	0.829–2.138	0.236	1.332	0.800–2.217	0.271
Tumor size (cm) (>4.3/≤4.3)	1.479	0.916–2.389	0.110	0.982	0.583–1.656	0.947
Differentiation degree (Well/Moderate/Poor)	1.092	0.793–1.503	0.590	0.969	0.692–1.356	0.853
T‐stage (T3 + T4/T1 + T2)	2.272	1.224–4.217	**0.009**	2.228	1.166–4.256	**0.015**
Lymph node metastasis (positive/negative)	2.636	1.565–4.439	**0.000**	3.628	2.020–6.517	**0.000**
NFIA (high/low)	3.450	1.908–6.240	**0.000**	3.388	1.801–6.371	**0.000**
NFIB (high/low)	1.310	0.802–2.139	0.281	1.210	0.715–2.048	0.477

OS, overall survival; DFS, disease‐free survival. The bold values indicated that the *P* value was smaller than 0.05.

### High NFIB expression correlates with lymph node metastasis and poor differentiation in EJA

**Figure 4:**
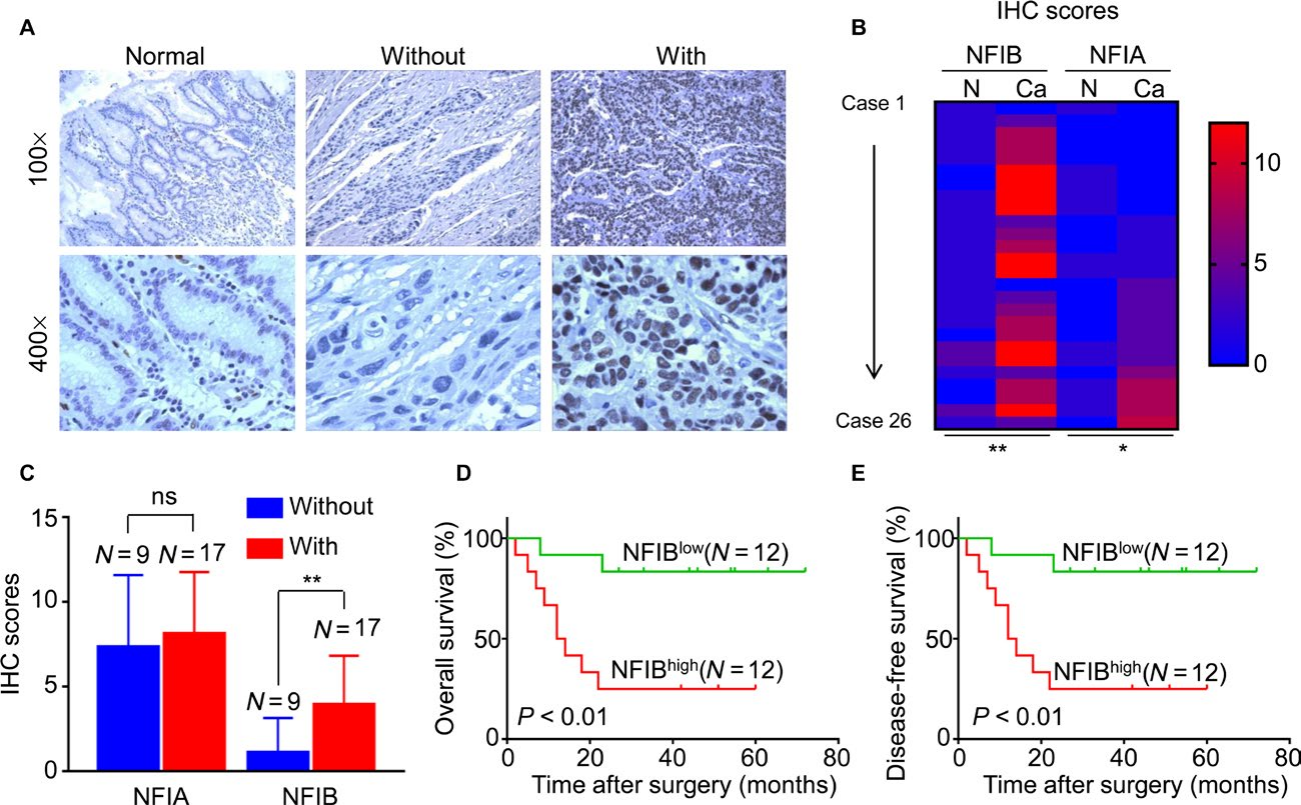
High NFIB expression predicts poor clinical outcomes of patients with esophagogastric junction adenocarcinoma (EJA). (A) Representative images showing the expression of NFIB in normal gastric epithelia and EJA tissues with or without lymph node metastasis. (B) Heat map showing the IHC scores of NFIA and NFIB in EJA tissues and corresponding normal esophageal epithelia. (C) IHC scores of NFIB in EJA tissues with or without lymph node metastasis. (D and E) The Kaplan–Meier survival analysis showing that EJA patients with high NFIB expression tend to have a shorter OS (C) or DFS (D) time. *P < 0.05; **P < 0.01.

The expression of NFIA and NFIB was then evaluated in EJA tissues from 26 patients, including 22 males and 4 females. The average age at diagnosis was 65.8. As shown in Figure 4A, NFIA and NFIB were expressed if any only in basal cells of normal gastric epithelial, mainly located in the nucleus. NFIB was highly expressed in EJA tissues. High expression of NFIB was found in cancerous tissues in 46.2% of the patients. The expression of both NFIA and NFIB was significantly higher than that in normal gastric epithelia (Fig. 4B). Chi‐square test revealed that high NFIB expression significantly correlated with lymph node metastasis (*P *=* *0.014) and advanced TNM stage (*P *=* *0.036) in EJA (Table 4). Additionally, NFIB was highly expressed in cancer tissues with lymph node metastasis in comparison with those without lymph node metastasis (Fig. 4C). However, there was no significant correlation between NFIA and clinicopathological features. Altogether, these results revealed that NFIB was highly expressed in EJA tissues and high NFIB expression correlated with lymph node metastasis and advanced TNM stage in EJA.

**Table 4:** Correlation between NFIA/NFIB expression and clinicopathological features in cancer tissues from 26 patients with esophagogastric junction adenocarcinoma

Clinicopathologic features	No. of patients (%)	NFIA expression status		*P* value	NFIB expression status		*P* value
		Low (*n* = 9) No. of patients (%)	High (*n* = 17) No. of patients (%)		Low (*n* = 14) No. of patients (%)	High (*n* = 12) No. of patients (%)	
Gender							
Male	22 (84.6)	9 (40.9)	13 (59.1)	0.263	11 (50.0)	11 (50.0)	0.598
Female	4 (15.4)	0 (0)	4 (100.0)		3 (75.0)	1 (25.0)	
Age							
≤66	12 (38.5)	3 (30.3)	9 (70.0)	0.429	6 (50.0)	6 (50.0)	1.000
>66	14 (61.5)	6 (37.5)	8 (62.5)		8 (57.1)	6 (42.9)	
Tumor size (cm)							
≤5.4	12 (26.9)	5 (57.1)	7 (42.9)	0.683	6 (50.0)	6 (50.0)	1.000
>5.4	14 (73.1)	4 (26.3)	10 (73.7)		8 (57.1)	6 (42.9)	
Differentiation degree							
Well	7 (26.9)	2 (28.6)	5 (71.4)	0.662	4 (57.1)	3 (42.9)	0.186
Moderate	6 (23.1)	3 (50.0)	3 (50.0)		5 (83.3)	1 (16.7)	
Poor	13 (50.0)	4 (30.8)	9 (69.2)		5 (38.5)	8 (61.5)	
T‐stage							
T2	3 (11.5)	0 (0)	3 (100.0)	0.407	3 (100.0)	0 (0)	0.193
T3	18 (69.2)	7 (38.9)	11 (61.1)		8 (44.4)	10 (55.6)	
T4	5 (19.3)	2 (40.0)	3 (60.0)		3 (60.0)	2 (40.0)	
Lymph node metastasis							
Negative	9 (34.6)	4 (44.4)	5 (55.6)	0.667	8 (88.9)	1 (11.1)	**0.014**
Positive	17 (65.4)	5 (29.4)	12 (70.6)		6 (35.3)	11 (64.7)	
Distant metastasis							
Negative	26 (100.0)	9 (34.6)	17 (65.4)	na	14 (53.8)	12 (46.2)	na
Positive	0 (0)	0 (0)	0 (0)		0 (0)	0 (0)	
TNM stage							
II	8 (30.8)	4 (50.0)	4 (50.0)	0.382	7 (87.5)	1 (12.5)	**0.036**
III	18 (69.2)	5 (27.8)	13 (72.2)		7 (38.9)	11 (61.1)	

Chi‐square test was used to evaluate the correlation between NFIA/NFIB expression and clinicopathological features. The bold values indicated that the *P* value was smaller than 0.05.

### High NFIB expression predicts poor outcomes of patients with EJA

We obtained follow‐up information from 24 patients with EJA, including 20 males and 4 females. The mean time was 33.7 months. The Kaplan–Meier analysis illustrated that high NFIB expression correlated with short OS (Fig. 4D; *P *<* *0.001) or DFS (Fig. 4E; *P *<* *0.001) time in patients with EJA, but NFIA did not correlate with OS or DFS time (Fig. S1A and B). These results demonstrated that high NFIB expression is of negative prognostic value in EJA.

## Discussion

Although much progress has been made in the last decades, the prognosis of both patients with ESCC and patients with EJA is poor. Better understanding of the pathological and molecular features of these two cancers would provide novel targets for the diagnosis and treatment of EC. The present study found that high NFIA is an independent prognostic risk factor in ESCC, while NFIB predicts poor outcomes of EJA. It is worth noting that the age of patients with EJA (65.8) was larger than that of patients with ESCC (60.8), and the tumor size of EJA (5.4 cm) was also larger than that of ESCC (4.0 cm).

Although the initial role of NFIs was demonstrated in the development of many organ systems, such as central nervous system 25 and lung 26, recent studies revealed that NFIs also play essential role in the context of cancer 7. NFIA mainly acts as a tumor‐promoting gene in glioma and ESCC 10, while NFIB exerts its oncogenic effect in SCLC, melanoma, breast cancer, and colon cancer and functions as a tumor suppressor in osteosarcoma, cutaneous squamous cell carcinoma, and NSCLC 11. Interestingly, Denny recently reported that chromatin in metastatic lesions exhibited a widespread increase in accessibility at gene distal regions that are enriched for NFI motifs, and NFIB regulates the expression of genes related to axon guidance, focal adhesion, and extracellular matrix–receptor interactions 13. Most recently, NFIB has been shown to promote proliferation of breast cancer cells in the absence of estrogen and inhibit the transcription activity of ER*α* 18. Consistent with the previous study that NFIA promotes growth of ESCC cells 11, we show here that NFIA is overexpressed in ESCC tissues, and high NFIA expression correlates with poor differentiation, lymph node metastasis, and advanced TNM stage in ESCC. It is worth noting that although NFIB was also overexpressed in ESCC, it is of no clinicopathological value in ESCC. On the other hand, NFIB was highly expressed in EJA, and high NFIB expression is of negative prognostic value in EJA. The small sample size of EJA is a main limitation of this work. Further work is needed to validate the role of NFIB in EJA using a large sample size. The differential roles of NFIA and NFIB reflect not only the distinct features of ESCC and EJA, but also the versatile functions of NFI family members.

The molecular mechanisms regulating the expression of NFIA and NFIB are still poorly known. Few studies showed that NFIA was targeted by microRNAs, including miR‐29a 10 and miR‐223 9. Another study demonstrated that activation of NF*κ*B signaling directly enhanced the transcription of NFIA in glioblastoma cells 27. The expression of NFIB was also regulated by microRNAs, such as miR‐372/373 28, miR‐153 29, 30, miR‐365 21, and miR‐124 31. In adult neural progenitors, the Pax6–BAF complex transcriptionally upregulated NFIB 8. Additionally, Drosha was recently reported to directly repress the transcription of NFIB independently of Dicer and microRNAs in adult neural stem cells 25. Estrogen receptors ER and PR might downregulate NFIB as it has been reported that the expression of NFIB was conversely associated with that of ER and PR 17. The molecular mechanisms by which NFIA and NFIB are upregulated in EC need to be illustrated. Moreover, how NFIA and NFIB exert their oncogenic roles in ESCC or EJA remains to be explored.

In conclusion, the present work revealed the clinicopathological and prognostic value of NFIA in ESCC and NFIB in EJA. High NFIA expression and high NFIB expression are associated with poor prognosis of patients with ESCC and patients with EJA, respectively. These results suggest that NFIA and NFIB might be novel markers for the diagnosis and treatment of ESCC and EJA, respectively.

## Conflict of Interest

The authors declare no conflict of interest.

## Supporting information


**Figure S1.** NFIA expression does not correlate with prognosis of patients with EJA.
