# Supplementary figures and images for: Prognostic significance of NFIA and NFIB in esophageal squamous carcinoma and esophagogastric junction adenocarcinoma
Source: Cancer Med. 2018 Mar 25;7(5):1756–65. doi: 10.1002/cam4.1434 (PMC5943462; doi:10.1002/cam4.1434)

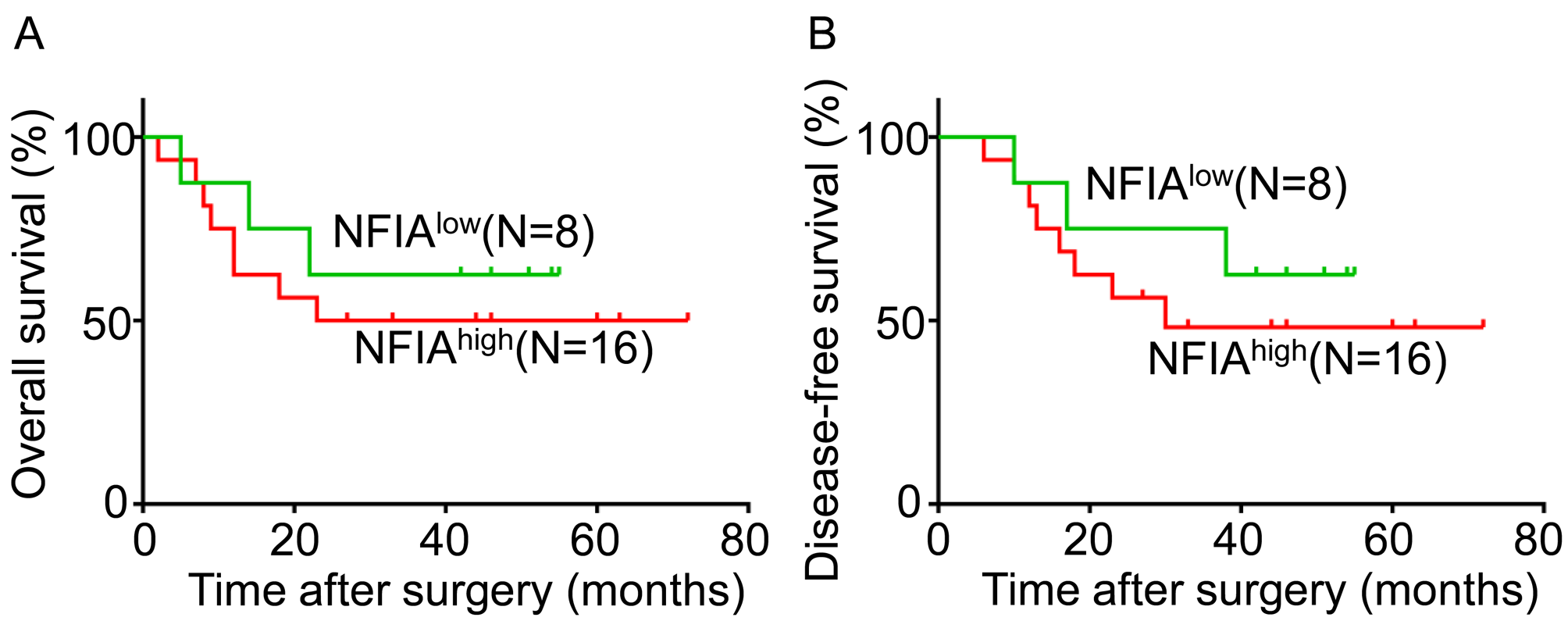

Supplement: Supplementary file 1 — Figure S1. NFIA expression does not correlate with prognosis of patients with EJA. [file CAM4-7-1756-s001.tif]
