# Supplementary material for: Prognostic significance of NFIA and NFIB in esophageal squamous carcinoma and esophagogastric junction adenocarcinoma
Source: Cancer Med. 2018 Mar 25;7(5):1756–65. doi: 10.1002/cam4.1434 (PMC5943462; doi:10.1002/cam4.1434)
Supplement: Supplementary file 2 [file CAM4-7-1756-s002.docx]

**Supplementary Figure 1. NFIA expression does not correlates with prognosis of EJA patients** A&B. Kaplan-Meier survival analysis showing that NFIA expression was correlated with neither OS (A) nor DFS (B) time.
